# Long‐Term Efficacy and Safety of Digital‐Single‐Operator‐Video‐Pancreatoscopy Guided Lithotripsy for Pancreatic Duct Stones

**DOI:** 10.1002/ueg2.70063

**Published:** 2025-06-06

**Authors:** Claudio C. Conrad, Mark Ellrichmann, Michiel Bronswijk, Schalk van der Merwe, Tobias Dertmann, Hadil Layka, Radhika Chavan, Sanjay Rajput, Pieter Jan de Jonge, Peter D. Siersema, Marianne Udd, Leena Kylanpaa, Philip Grunert, Gilbert Rahe, Christoph Schramm, Jassin Rashidi‐Alavijeh, Marco J. Bruno, Torsten Beyna, Christian Gerges

**Affiliations:** ^1^ Interdisciplinary Endoscopy Department for Internal Medicine University Hospital Schleswig–Holstein Kiel Germany; ^2^ Department of Gastroenterology and Hepatology University Hospital Leuven Leuven Belgium; ^3^ Imelda General Hospital Bonheiden Belgium; ^4^ Department of Internal Medicine and Gastroenterology Evangelisches Krankenhaus Düsseldorf Düsseldorf Germany; ^5^ Ansh Clinic Ahmedabad India; ^6^ Department of Gastroenterology and Hepatology Erasmus MC University Medical Center Rotterdam the Netherlands; ^7^ Department of Gastrointestinal Surgery Helsinki University Hospital Helsinki Finland; ^8^ Department for Interventional Endoscopy University Hospital Essen Essen Germany; ^9^ Medical Clinic II Department of Gastroenterology, Hepatology, General Internal Medicine and Infectious Diseases Helios Clinic Krefeld Krefeld Germany

**Keywords:** chronic calcifying pancreatitis, digital video pancreatoscopy, ductal decompression, electrohydraulic lithotripsy, extracorporeal shock wave lithotripsy, pain management, pancreatic stones, quality of life

## Abstract

**Introduction:**

Ductal decompression has become the main approach for treating patients with symptomatic chronic calcifying pancreatitis and signs of ductal hypertension. Digital single operator video pancreatoscopy (dSOVP) has shown high success rates when compared with more established techniques such as extracorporeal shock wave lithotripsy. However, there is still limited evidence on long‐term clinical success and quality of life.

**Methods:**

Patients with chronic calcifying pancreatitis who underwent digital single operator video pancreatoscopy guided electrohydraulic lithotripsy (EHL) of pancreatic duct stones with initial technical and clinical success were recruited for this retrospective, multicenter cohort study. Persistence of clinical success (defined as pain reduction > 50% in numerical rating scale [NRS]) as well as postinterventional quality of life (QOL) were retrospectively evaluated by database analysis and with QOL using the Mental and Physical Condition Scores (MCS, PCS).

**Results:**

A total of 58 patients were included in the long‐term follow‐up conducted over 24 months. Significant and sustained pain relief was reported in 70.7% of patients (*n* = 41) at month 3; this effect persisted until month 24. MCS decreased from 50.36 ± 13.3 at baseline to 49.75 ± 11.1 at month 12 with no statistically significant difference (data available for 42 patients, *p* = 0.15). Similarly, the PCS showed no significant improvement, remaining constant at 44.9 ± 9.8 at baseline and 44.9 ± 10.8 at month 12 (*p* = 0.1). The overall adverse event rate was 26% (11 patients), primarily consisting of mild to moderate pancreatitis (*n* = 9, 22%).

**Conclusions:**

Digital single operator video pancreatoscopy guided lithotripsy was shown to be safe and effective in a long‐term follow‐up regarding pain control but had no significant influence on QOL. Complete stone removal seems to be the key point for long‐term clinical success.

## Graphical abstract

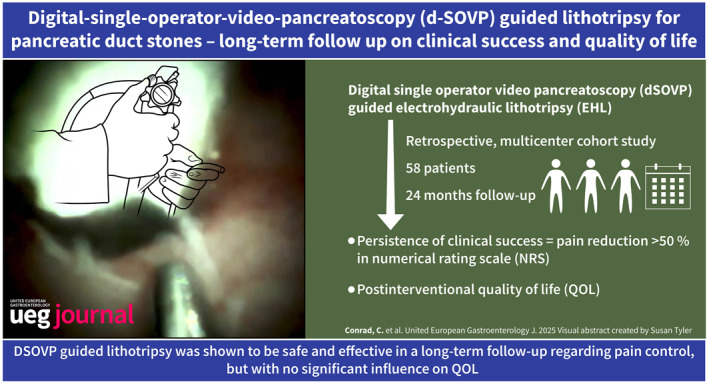

1

**Summary**

- Summarize the established knowledge on this subject
  ◦ Ductal decompression has become the main approach for the treatment of chronic calcifying pancreatitis
  ◦ Digital single operator video pancreatoscopy with electrohydraulic lithotripsy provides high success rates in achieving complete duct clearance
  ◦ Limited evidence on long‐term clinical success and quality of life
- What are the significant and/or new findings of this study?
  ◦ Digital single operator pancreatoscopy with electrohydraulic lithotripsy is safe and effective in a long‐term follow‐up
  ◦ Complete duct clearance seems to be the key point for long‐term clinical success
  ◦ The quality of life is neither positively nor negatively influenced by the intervention

## 2 Introduction

Abdominal pain is the most frequent and debilitating symptom in chronic calcifying pancreatitis (CCP) severely impairing patients’ impacts quality of life (QOL). A key driver of this pain is ductal hypertension often caused by pancreatic duct (PD) stones. PD stones have been shown to be associated with pain exacerbations and recurrent flares of pancreatitis [1, 2, 3, 4]. Given this pathophysiological link, the primary aim of endoscopic management in CP‐related pain should be the relief of ductal hypertension through PD drainage removal of obstructing stones and the management of strictures [5]. Current guidelines recommend extracorporeal shock wave lithotripsy (ESWL) as the first‐line treatment of PD‐stones [5, 6]. However, its limitations include lack of availability, operator dependent outcomes, need for multiple ESWL sessions, and inability to treat underlying strictures [7].

Since the introduction of digital single operator video cholangio‐/pancreaticoscopy (dSOVP) in 2015, dSOVP has become an emerging alternative to standard endoscopic procedures for the treatment of CCP, with reported success rates between 88% and 100% [8, 9, 10, 11, 12]. A recently published prospective study was even able to demonstrate even superiority over ESWL [13]. However, while reported technical and short‐term clinical success rates seem promising, there is still limited evidence regarding long‐term success and effects on QOL.

Our aim was to investigate the long‐term clinical success and QOL of dSOVP guided PD‐stone electrohydraulic lithotripsy (EHL) with complete duct clearance (CDC) in patients with CCP.

## 3 Material and Methods

**FIGURE 1:**
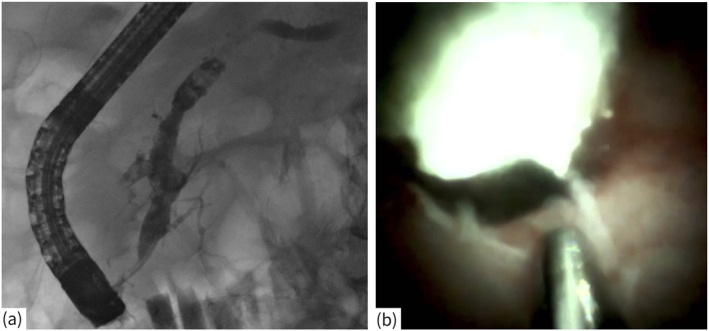
(a) Chronic calcifying pancreatitis with PD stones; (b) dSOVP with lithotripsy probe and PD stone.

In this retrospective, international, multicenter study, we recruited sequentially treated patients who underwent dSOVP‐guided lithotripsy with EHL (Figure 1) with initial technical and clinical success at five tertiary referral centers between 2015 and 2022. All dSOVPs were performed with Spyglass DS System (Boston Scientific, Natick, Mass, USA) and EHL either with Autolith (Boston Scientific, Natick, Mass, USA) or Walz (Walz Elektronik GmbH, Rohrdorf, Germany) probe. The settings of the probe were set by the examiner.

Inclusion criteria included: (1) imaging proven PD‐stones using a combination of EUS, abdominal MR and/or CT, (2) upstream PD dilation, and (3) pain attributable to CCP.

All patients had EUS and CT and/or MRCP prior to dSOVP. Other treatment options (surgery, ESWL or Endoscopic Retrograde Cholangiopancreatography [ERCP]) had already been attempted, but were deemed less suitable than dSOVP, or were refused by the patient.

Central institutional review board approval was attained at the Medical Faculty of the University of Schleswig–Holstein, Campus Kiel, Kiel, Germany (reference number: D443/23) and at each individual center. Written consent was obtained from all participating patients. Patients were discussed in an interdisciplinary pancreatic expert board in the predefined clinic before each procedure.

### 3.1 Outcomes

The persistent clinical success after intervention with a decrease of > 50% in a Numerical Rating Scale (NRS) in a long‐term follow‐up over 24 months was defined as the primary endpoint of this study.

The secondary endpoint comprises the change in quality of life, including in the long‐term follow‐up phase. Patients were interviewed using a standardized questionnaire adapted from the SF‐12, complemented by questions about specific and general symptom burden and average pain levels. When determining the QOL, the two subscores mental condition score (MCS) and physical condition score (PCS) were also included. These were determined over time and compared with the baseline for each case.

Persistence of clinical success was defined as a sustained post‐interventional reduction of pain levels on NRS for pain of > 50%. The NRS is a segmented numeric version of the visual analog scale (VAS) with numerical reflection of pain intensity (score 1–10).

### 3.2 Follow‐Up

All patients underwent a systematic follow‐up. This included determining the current pain level and completing the QOL questionnaire. After the initial procedure with dSOVP combined with electrohydraulic lithotripsy for stone fragmentation with successful complete ductal clearance, all patients were rescheduled for a subsequent follow‐up. Follow‐ups took place after 6, 12, and 24 months and were performed at the outpatient clinic or by telephone calls, and database analysis.

### 3.3 Statistical Analysis

Quantitative data were summarized by means, medians, range, and interquartile range (IQR) as appropriate. Statistical significance was tested with a two‐tailed *t*‐test or Fisher's exact test. A *p* < 0.05 was considered statistically significant. GraphPad prism 9 and MS excel and orthotoolkit.com/SF‐12 were used as statistical tools.

## 4 Results

**FIGURE 2:**
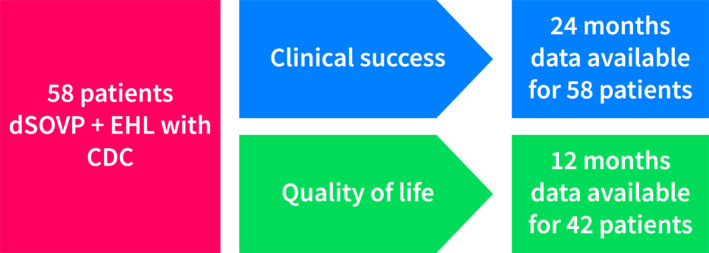
Flowchart of the retrospective analysis.

A total of 58 patients (37.9% female) were included in the study, with a follow‐up for 24 months to determine clinical success. Of these patients, quality of life data was available for 42 patients up to 12 months of follow‐up (Figure 2).

Mean age of the patients was 59.2 ± 12.8 years with a body mass index (BMI) of 24.51 kg/m2 ± 4.06. The etiology of CCP was toxic/alcoholic (43.1%) or idiopathic (32.8%) in the majority of cases. At baseline, 54 (93.1%) patients had a dilated duct > 5 mm. Most patients were found to have a single stone (*n* = 29; 50%) with the head as the leading localization (*n* = 40; 69%). Detailed patient characteristics are presented in Table 1.

**TABLE 1:** Patient characteristics.

Number of patients [N]	58
Female [N] (%)	22 (37.9)
Age [years] (mean ± SD)	59.2 ± 12.8
BMI (Mean ± SD)	24.51 ± 4.06
ASA‐classification [N] (%)	
ASA 1	11 (19)
ASA 2	31 (53.4)
ASA 3	9 (15.5)
ASA 4	2 (3.4)
unknown	5 (8.6)
Etiology [N] (%)	
Alcohol	25 (43.1)
Idiopathic	5 (8.6)
Auto‐immune	1 (1.7)
Other	8 (13.8)
unknown	19 (32.8)
PD interventions (mean ± SD)	1.3 ± 0.6
Dilated duct > 5 mm	54 (93.1)
Number of stones [N] (%)	
1	29 (50)
2	12 (20.7)
3	5 (8.6)
4	5 (8.6)
> 5	7 (12.1)
Stone localization [N] (%)	
Head	40 (69)
Genu	12 (20.7)
Corpus	6 (10.3)

### 4.1 Outcomes

**FIGURE 3:**
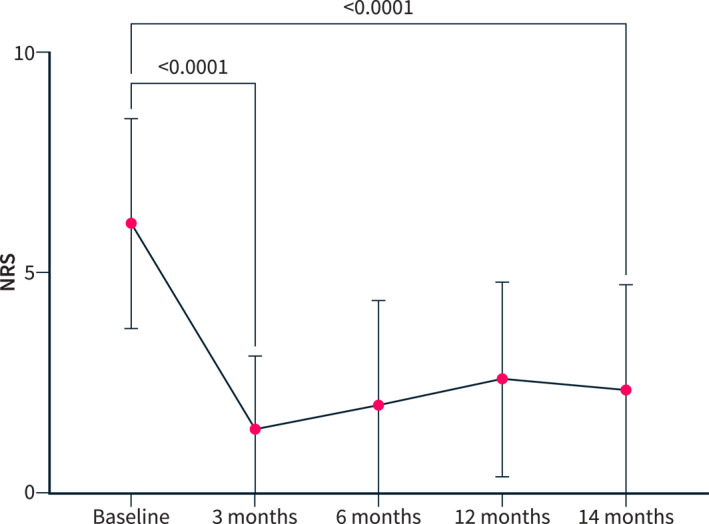
NRS follow‐up.

Fifty‐two patients (89%, 7%) had a clinical success with a decrease in the NRS of ≥ 50%, from the baseline mean of 6.07 (± 2.34) to 1.07 (± 0.85) at month 3 (*p* < 0.0001). The NRS in all 58 patients decreased from a mean of 6.16 (± 2.39) at baseline to 1.47 (± 1.68) at month 3 (*p* < 0.0001; Figure 3).

**FIGURE 4:**
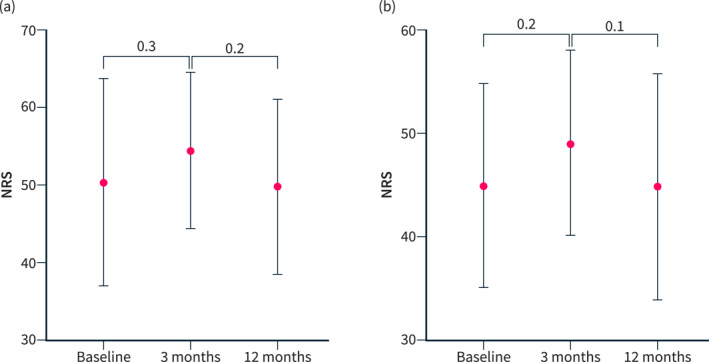
(a) MCS quality of life subscore follow‐up; (b) PCS quality of life subscore follow‐up.

The Mental Condition Score (MCS) fell non‐significantly from 50.36 (± 13.27) at baseline to 49.75 (± 11.11) at month 12 (*p* = 0.15). The Physical Condition Score (PCS) was 44.97 (± 9.79) at baseline and showed no significant improvement over 12 months 44.85 (± 10.81) (*p* = 0.1; Figure 4).

The median follow‐up duration was 24 months.

Long‐term clinical success was seen in 41 patients (70.7%) with a decrease in the NRS of ≥ 50% from baseline to month 3, which persisted until month 24.

In all patients, the NRS was 2.35 (± 2.41) at month 24 (Figure 3).

The overall adverse event rate was 11 (26%) based on available data from 41 patients. Nine patients (22%) developed post‐ERCP pancreatitis (8 mild, 1 moderate), representing the most frequent adverse event, while 2 patients (4.9%) had post‐procedure fever. CDC was achieved in 46 patients (79%) in a single session with a mean of 1.3 ± 0.7 PD interventions in all patients. 43 (74%) patients underwent therapeutic stenting, which was performed at the investigator’s discretion independently of CDC. The stents were removed after a mean of 3.6 ± 1.5 months.

## 5 Discussion

The main goal of endoscopic therapy in patients with obstructive CCP should be PD de‐obstruction with complete removal of PD stones and treatment of any remaining ductal strictures, as PD obstruction is regarded as the main driver of symptoms and complications. A growing body of evidence suggests that dSOVP lithotripsy is a promising treatment option when standard approaches, such as ERP or ESWL, have failed to clear the pancreatic duct [9, 13, 14, 15, 16, 17, 18, 19, 20]. DSOVP has a high technical and clinical success rate with ductal clearance reported in between 88% and 100%, with low adverse event rates between 0% and 30% [9, 10]. The largest conducted study to date by Bremer Gutierrez et al. in 2019 with 109 patients showed that dSOVP performed with either EHL or laser lithotripsy is safe and effective [11]. Single session ductal clearance was achieved in 77.1% of patients with EHL with an AE rate of 8.5%. These findings align with the rates observed in our study. For this reason, many expert centers currently favor dSOVP, reserving ESWL for selected cases only. It is also assumed that patients with PD stones tend to have harder stones, which can be treated better with EHL than with ESWL. This is supported by a urological study which shows that the success of ESWL depends on the density of the stones [21] but is not yet proven in pancreatic stones.

The ESCAPE trial, which compared early surgery with an endoscopy‐first approach for pain reduction in chronic pancreatitis, showed a superiority of surgical therapy [22]. The study demonstrated complete or partial pain relief in 58% of surgically treated patients compared with 39% when patients were managed endoscopically. However, this difference was not statistically significant. A further limitation of the study is the relatively low technical success rate in the endoscopic arm, where ductal clearance was achieved in only 24 of 44 patients (55%). Notably, among patients who achieved complete ductal clearance with endoscopy, pain relief outcomes were comparable to those in the surgical group. Thus, CDC seems to be a key prognostic parameter, consistent with our findings. In addition, endoscopic therapy in this study was performed using conventional endoscopic stone removal techniques or by pancreatic stenting. Furthermore, ESWL was not consistently applied. The authors emphasized that higher ductal clearance rates could probably be achieved with novel endoscopic procedures such as dSOVP, which was not yet largely available at the time the study was performed.

Other older studies have also explored the hypothesis of surgical superiority over endoscopic treatment. Díte et al. in 2003 reported similar findings compared to those in the ESCAPE trial in a trial with 72 randomized (and a total of 140) patients [23]. After a 5‐year follow‐up period, complete pain absence was more frequent after surgery (34% vs. 15%), while the rate of partial pain relief was similar in both groups (52% vs. 46%). However, neither EWSL, nor subsequent endoscopic therapy was performed for a recurrence of symptoms.

These shortcomings were addressed by Cahen et al. in 2007 and 2011 [24, 25] by comparing surgical drainage procedures to endoscopic drainage. A total of 39 symptomatic CP patients with complex pathologic features (79% presenting with a combination of strictures and stones) underwent randomization to endoscopic drainage or operative pancreaticojejunostomy (Partington‐Rocelle). Over a mean follow‐up of 79 months, surgery was deemed superior, though statistical significance was achieved only for combined partial and complete pain relief. This study was limited by a small sample size and lack of statistical power. Moreover, all patients already had advanced CCP and an opioid‐dependent course. The endoscopic success rate was very low (55%), with most failures being explained by pancreatic duct access issues. Collectively, in recent randomized trials, optimal surgical techniques have been compared to suboptimal endoscopic techniques not aiming for complete ductal clearance, adequate management of stricutres, or alternatives when ductal access fails. The use of therapeutic EUS may facilitate successful ductal access when an ERP has failed, for example by performing a rendezvous or EUS‐guided pancreatogastrostomy [26].

In our study, which to our knowledge represents the largest long‐term follow‐up cohort of CCP patients treated with dSOVP guided EHL to date, we showed a persistent clinical success of 71% of patients after 24 months. This indicates that dSOVP is at least comparable to the outcomes of historical surgery cohorts, ESWL, or other endoscopic procedures in terms of pain control.

Our study, along with the above discussed studies, also shows that complete ductal clearance might be a good prognostic factor for clinical success and therefore should be the primary therapeutic goal at least in the presence of occluding PD stones.

With regard to quality of life, our study is the first to have data on long‐term follow‐up after intervention using dSOVP. We were unable to demonstrate QOL improvement in either PSC or MCS. It is therefore reasonable to assume that pain alone might not be the sole determinant of poor QOL in CCP. CCP is a complex disease with many complications that impact QOL, the ability to work, which induce psychosocial stress that may be reflected in health questionnaires. In addition, patients often have several comorbidities, such as cardiovascular, pulmonary or metabolic diseases, which may also impact QOL.

### 5.1 Limitations

Despite the fact that our study provides data on long‐term outcomes following dSOVP, our study also has some limitations. This includes its retrospective design, inherently increasing the risk of selection bias and confounding factors. The cohort further lacked a control group and is likely to have selection bias. Future studies should focus on further improving patient selection, as well as comparing currently available endoscopic therapy (including dSOVP and EUS‐assisted access) with surgery.

## 6 Conclusion

In conclusion, in our study dSOVP guided lithotripsy was found to be safe and effective during long‐term follow‐up. Complete stone removal seems to be the key point for long‐term clinical success. CDC therefore appears to be decisive for the success of the intervention. Prospective studies need to clarify to what extent dSOVP will replace ESWL and to what extent endoscopic therapy with dSOVP compares with surgery.

## Conflicts of Interest

Marco Bruno was with Boston Scientific as consultant, support for industry and investigator‐initiated studies, Cook Medical as consultant, support for industry and investigator‐initiated studies, Pentax Medical as consultant, support for investigator‐initiated studies, Mylan provided support for investigator‐initiated studies, AMBU as consultant, support for investigator‐initiated studies. ChiRoStim provided support for investigator‐initiated studies. Pieter Jan De Jonge, was with Boston Scientific as consultancy fees, Cook Medical as consultancy fees, Fujifilm Europe as consultancy fees. Peter Siersema was with Pentax—Japan as grant/research support, MicroTech—China as grant/research support, Fuji Film—Japan as grant/research support. Gilbert Rahe was with Boston Scientific, as lecture fees, Microtech, as lecture fees. Mark Ellrichmann was with Boston Scientific as study fees, consultancy honoraria, and travel expenses, Microtech as study fees, consultancy honoraria, and travel expenses.

## Data Availability

The data that support the findings of this study are available on request from the corresponding author. The data are not publicly available due to privacy or ethical restrictions.
